# Eye movement and reading behavior in older adults with dementia

**DOI:** 10.1186/s12877-026-07030-8

**Published:** 2026-01-29

**Authors:** Ayano Inuyama, Motoshi Ouchi, Shigeki Hirano, Sayuri Suwa

**Affiliations:** 1 Department of Community Health Nursing, Graduate School of Nursing, Chiba University, 1-8-1 Inohana, Chuo-ku, Chiba, 260-8672 Japan; 2 Department of Health Promotion in Nursing and Midwifery, Graduate School of Nursing, Chiba University, 1-8-1 Inohana, Chuo-ku, Chiba, 260-8672 Japan; 3 Department of Neurology, Graduate School of Medicine, Chiba University, 1-8-1, Inohana, Chuo-ku, Chiba, 260-8670 Japan

**Keywords:** Dementia, Eye movements, Eye tracking, Reading, Japanese, Older adults

## Abstract

**Background:**

With the rising number of patients with dementia, it is essential to develop support strategies that incorporate visual information. This study aimed to clarify differences in eye movement patterns when older adults with dementia read documents of varying difficulty, in order to identify strategies for enhancing readability and supporting comprehension.

**Methods:**

Eye movements were measured using an eye tracker while nine older adults with dementia read documents. Each document had an original version and a revised version, designed to improve readability. Data were compared across document types, original versus revised versions, and aloud versus silent reading. Video recordings were qualitatively analyzed.

**Results:**

In the comparison of document types, “skipping over” (*p* = 0.046) and “misreading” (*p* = 0.050) significantly decreased in documents of lower difficulty. No significant difference was observed between aloud and silent reading. However, among participants who read aloud, the revised version showed significant reductions in reading time (*p* = 0.028), fixation count (*p* = 0.046), and regression (*p* = 0.046). Qualitative analysis yielded three major categories: Response to caregiver support, Reading strategies, and Challenges in using support.

**Conclusion:**

The revised documents improved eye movement patterns, suggesting enhanced readability. In addition to text modifications, individualized caregiver support was critical for comprehension. These findings can inform the design of written information for older patients with dementia.

**Supplementary Information:**

The online version contains supplementary material available at 10.1186/s12877-026-07030-8.

## Background

More than 57 million people worldwide have dementia, with nearly 10 million new cases diagnosed annually [1]. Dementia is characterized by progressive impairment of higher cognitive functions, such as memory, attention, executive function, and language, which interfere with daily living activities [2]. In this context, supporting the daily lives of patients with dementia is increasingly important. One approach is Talking Mats, a low-tech communication framework that facilitates emotional expression by arranging relevant images on a visual scale [3]. Studies report that patients with dementia and their caregivers feel more engaged in discussions about daily life management when using Talking Mats compared with standard communication methods [3]. These findings indicate that visual information can effectively support communication in patients with dementia. Text, a form of visual information, is widely used in medical decision-making and everyday contexts, including treatment instructions, medication labels, contracts, traffic signs, and product descriptions. However, research has shown that visually complex or low-frequency kanji negatively affect reading comprehension in older adults with dementia [4]. Thus, patients with dementia may struggle with reading.

Written information is processed through visual perception [5]. Eye movement analysis during document reading, often measured with eye trackers, provides an objective and reliable method of evaluating text processing [6]. Reading involves fixations (stable gaze) and saccades (rapid jumps between words), with information acquired only during fixations [7]. The visual field during reading is divided into the fovea, parafovea, and periphery, with the fovea providing sharp acuity. Skilled readers move their eyes to align the fovea with words requiring clear resolution [6]. Individuals with lower reading comprehension typically show longer fixations, shorter saccades, and more fixations overall [6]. Abnormal eye movements are not considered the cause of reading disorders but reflect the difficulties faced by dyslexic readers [8].

Individuals with dementia exhibit distinctive eye movement patterns compared with controls. In digit reading tasks, they demonstrate shorter saccade duration and amplitude, and they require more time to complete tasks [9]. In clock-reading tasks, they display reduced fixation on relevant areas, delayed fixation onset, longer average fixation durations, and shorter saccade amplitudes [10]. When the frontal lobe is affected, the ability to suppress reflexive saccades in response to visual obstructions may be impaired, resulting in large saccades occurring during fixation [11]. These patterns resemble those of readers with low comprehension, suggesting that they may impair document reading in individuals with dementia. Prior studies have shown that, during document reading, patients with dementia, compared with older adults without dementia, display increased fixation counts, more reverse saccades, higher rates of skipped words [12], slower reading speed, significantly longer fixation durations, more forward saccades per line, and more backward saccades [13]. Such abnormalities in patients with Alzheimer’s disease may serve as early indicators of reading impairment before subjective complaints or measurable deficits appear in reading tests [13]. Eye movement assessment may therefore be valuable for early diagnosis and monitoring disease progression. Nevertheless, limited attention has been given to document content, difficulty, design, and support strategies for individuals with dementia.

Although patients with Alzheimer’s disease often retain reading comprehension, they tend to read slowly and display irregular eye movements [13]. To support their reading ability, it is essential to account for these eye movement characteristics and design accessible texts. However, few studies have addressed strategies to improve document comprehension in older adults with dementia. One study comparing reading comprehension under three display conditions (standard text, one word, and two words) reported modest accuracy improvements in patients with Alzheimer’s disease under the one- and two-word conditions [14]. Another study examined how patients with dementia experienced reading classical novels in group settings and identified helpful modifications for supporting memory impairment, including providing a cast of characters, reducing text length, and repeating references [15]. Building on these findings, this study aimed to clarify differences in eye movement data when older adults with dementia read documents of varying difficulty. This study is exploratory in nature and aims to obtain foundational knowledge for future research. To supplement the eye-tracking data, video recordings of reading sessions were analyzed to capture additional details not evident from eye movements alone.

## Methods

### Participants

Participants included individuals aged ≥ 65 years diagnosed with dementia. All participants were literate Japanese, as the study required assessment of “eye movements” in response to viewing and reading Japanese text. Recruitment was conducted through daycare service facilities and residential facilities that routinely care for older adults with dementia (hereafter referred to as “nursing facilities”) and through the Dementia Medical Care Center, Chiba University Hospital (hereafter referred to as “the hospital”). Eligibility criteria included the ability to use the eye tracker, an eye movement measurement device; the ability to read text through the tracker’s lens; absence of visual field deficits; and sufficient verbal communication ability with the researcher. The tracker lenses could be adjusted in 0.5 diopter increments within a range of − 8.0 to + 3.0.

This study included nine participants. Participant characteristics are summarized in Table 1. Five participants were assessed using Document 1, and four using Document 2. The average age was calculated based on questionnaire responses. For participants who selected “70–74 years,” the median value of 72 was used.

**Table 1:** Summary of participant characteristics

		All participants (*n* = 9)	Document 1 (*n* = 5)	Document 2 (*n* = 4)
Age	75–79	6	4	2
	80–84	1	0	1
	85–89	1	1	0
	≥ 90	1	0	1
Calculated average age	Mean ± SD	80.3 ± 5.59	79.0 ± 4.47	82.0 ± 7.07
Sex	Male	5	4	1
	Female	4	1	3
Dementia severity (CDR)	Mild (%)	5 (55.6)	3 (60.0)	2 (50.0)
	Moderate (%)	4 (44.4)	2 (40.0)	2 (50.0)
Education(years)	Mean ± SD	13.3 ± 2.12	14.0 ± 2.45	12.0 ± 0.00

*CDR* Clinical Dementia Rating

### Data collection

#### Eye movements

Eye movements during document reading were measured using the Tobii Pro Glasses 2 (sampling rate: 100 Hz), with data analyzed in Tobii Pro Lab (version 1.108). As with other eye trackers, calibration was required before measurement. Calibration determines the precise point of gaze by recording eye data while the participant fixates on a researcher-designated point. The number of calibration points varies by device; for example, Nagarajan et al. used the EyeLink 1000 Plus (SR Research Ltd.) with a 5-point calibration [16]. In contrast, the Tobii Pro Glasses 2 requires only a single-point calibration [17], which was employed in this study. Each participant completed two measurements, one with the original document and one with the modified version, described below. Measurements were separated by a 10–15-minute break. Participants were free to choose their reading method (aloud or silent) and document position (handheld or on a desk).

#### Original and modified versions of documents

The documents used for eye movement were selected to reflect materials that participants were likely to see or read in daily life. Specifically, they were documents available at the recruitment sites (hospital or nursing home) and included relevant content: one excerpted from a study authored by a researcher not involved in this study (Document 1), and another created by facility staff to inform users of their plans for going out (Document 2). The document type varied by study site: participants at the hospital read Document 1, whereas those at the nursing facility read Document 2. Original and modified versions were written horizontally in Japanese, incorporating kanji, hiragana, katakana, and numbers. The criteria for creating the modified documents were based on findings from previous studies on characters [18, 19] and aphasia-friendly formats [20, 21]. Revisions included adjustments to font, font size, character spacing, spacing, kanji notation, and number of characters per sentence. The modified documents (Documents 1 and 2) used a Mincho font, 14-point font size, 1-point character spacing, and 21-point line spacing. The document size and number of pages remained unchanged from the original versions; the original and revised versions of Documents 1 and 2 were presented on a single A4 sheet to eliminate the influence of page turning. Document 1 was revised by reducing characters from 1,056 to 955, increasing lines from 29 to 37, and reducing kanji from 421 to 260. Document 2 was revised by reducing characters from 400 to 375, increasing lines from 17 to 18, and reducing kanji from 159 to 117. In both documents, the modifications decreased the number of characters and kanji while increasing the number of lines. When creating the revised versions, care was taken to preserve the amount of content. To confirm this, an independent third party read the original and revised versions and verified that there was no difference in the meaning conveyed. Optimal letter and line spacing values were determined using the Database of Sensory Characteristics of Older Persons and Persons with Disabilities [22]. Readability values were assessed using the Japanese text readability measurement system “jReadability” [23], an online tool in which higher scores indicate greater ease of reading. Readability values improved from the original to the modified versions in both documents: Document 1 increased from 1.76 to 3.5, and Document 2 from 3.09 to 3.67. The results confirmed enhanced readability in the modified versions.

“Readability” has been defined as “the ease with which readers can read and understand a given text” [24] and as “the totality of all elements within a printed text (including interactions) that affect the extent to which a reader group can understand the text, read it at an optimal speed, and maintain interest in it” [25]. Slattery et al. evaluated legibility using total reading time, number of fixations, and average fixation duration [26]. Based on these references, the present study defines readability as the ability to read fluidly without disruption when attempting to understand the content, regardless of whether comprehension is achieved.

#### Interviews

Each interview survey lasted 10–20 min and was conducted using a semi-structured format with pre-prepared questions. Since interviews were conducted after the original and modified eye movement measurements, each participant completed two interviews. The interview questions were developed specifically for this study. In the first interview, the researcher asked questions such as, “How difficult was it when reading documents?” and “How do you manage when you encounter such difficulties?” With the participant’s permission, the interview was either audio recorded or documented in written notes. Further details are provided in the supplementary materials (see Supplementary Materials 1).

### Data collection period

July 2019 to February 2020.

### Method of analysis

As noted above, eye movements during text reading consist of four components: saccade, fixation, backward movement, and line shifting [27]. In this study, the analysis focused on total reading time, mean fixation time, number of fixations, and number of backward movements. In Japanese horizontal text, line shifting refers to the leftward gaze shift from the end of one line to the beginning of the next. This differs from rereading behavior, defined as moving backward within the text [27]. However, in practice, it was difficult to distinguish backward movement from line shifting, since leftward shifts included movements such as from the end of a line to its middle, from the middle to the beginning, as well as standard line shifts. Therefore, all such leftward gaze shifts were categorized as backward movements in this study.

Based on video recordings from the eye tracker, analyses were conducted to examine how participants read the documents using quantitative and qualitative approaches. For the quantitative analysis, instances of reading aloud were counted, including occasions when participants became stuck, rereading/reading twice, reading one character at a time/inserting pauses, skipping over, reading in interrogative manner, or misreading. These patterns were then analyzed statistically. For the qualitative analysis, the thematic analysis method [28] was employed. Verbal and non-verbal behaviors of participants during document reading were transcribed and thoroughly reviewed to create initial codes. Major categories were then constructed based on the similarity of meaning content within these codes. Interview data were incorporated as supplementary information, for example, to better understand participants’ background characteristics.

Definitions of Japanese-specific pronunciation methods are provided below. Japanese consists of kanji, which are logographic characters, and two syllabaries, hiragana and katakana. Kanji often have multiple readings [29]. Words written in kanji were originally borrowed from Chinese; the original Chinese pronunciations are called on-reading, whereas the native Japanese pronunciations are called kun-reading [30]. Thus, the same character can have different pronunciations. For example, the kanji character “犬” (dog) is read as ”ken” in on-reading and “inu” in kun-reading. In addition to the variability of kanji, numbers can also be read in multiple ways. For instance, “100” may be read as “one hundred” or as “one zero zero.” Japanese speakers select the appropriate reading depending on the context. In this study, the term “the reading” refers to the selection of an appropriate pronunciation from among these alternatives.

Statistical analysis was performed using IBM SPSS Statistics (ver. 28), and *p* < 0.05 was considered statistically significant. P-values were rounded to third decimal places (e.g., *p* = 0.0495 was reported as *p* = 0.050). Mann–Whitney U test and Wilcoxon signed-rank test were used.

### Ethical considerations

The researchers conducted this study in accordance with the Declaration of Helsinki. All participants received a plain-language explanation of the study’s purpose, methods, and ethical considerations and provided written informed consent before participation. When participants were unable to sign, proxy consent was obtained from a family member or caregiver. Data collection considered the physical condition of participants with dementia on the day of the study, and willingness to participate was confirmed by ensuring no refusal was expressed verbally, through facial expressions, or through behavior. This study was approved by the Chiba University Graduate School of Nursing Ethics Review Committee (approval no.: 30–84).

## Results

### Measured eye movements and reading characteristics

Six participants chose to read aloud, and three chose to read silently. For Documents 1 and 2, three participants each chose to read aloud. Although this study does not directly confirm that participants who selected silent reading fully comprehended the documents, fixating on target characters represents the primary step required for reading. Eye-tracking data indicated that participants who read silently did fixate on the characters. Furthermore, in post-task interviews, participants who selected silent reading made comments such as, “The original text had small characters, but typical documents are about this size,” and “There are a lot of technical terms, aren’t there?” Based on these observations, we interpreted that participants who chose silent reading were also engaging in comprehension of the documents. Table 2 presents the results of eye movement analysis when comparing Documents 1 and 2 (top row) and the characteristics of participants’ reading aloud (bottom row). No significant differences were observed between Documents 1 and 2 in either the original or modified versions. However, in both versions, Document 2 showed lower average values for reading time, fixation count, and regression compared with Document 1. Analysis of reading-aloud behaviors indicated that, in Document 2 compared with Document 1, “skipping over” (*p* = 0.046) and “misreading” (*p* = 0.050) were significantly reduced in the original version, whereas “reading one character at a time or inserting pauses” (*p* = 0.046) was significantly reduced in the modified version.

**Table 2:** Comparison between Documents 1 and 2

	Original version				Modified version		
	Document 1 (*n* = 5)	Document 2 (*n* = 4)			Document 1 (*n* = 5)	Document 2 (*n* = 4)	
	Mean ± SD	Mean ± SD	P-Value		Mean ± SD	Mean ± SD	P-Value
Reading time (s)	316.86 ± 159.82	174.28 ± 25.16	0.142	Reading time (s)	224.64 ± 104.11	113.83 ± 2.71	0.142
Fixation count	648.00 ± 490.04	362.75 ± 208.01	0.462	Fixation count	557.60 ± 469.70	196.00 ± 86.74	0.142
Regression	192.00 ± 155.32	130.50 ± 89.68	0.624	Regression	169.80 ± 143.30	67.00 ± 36.14	0.086
Average fixation duration (s)	0.31 ± 0.16	0.20 ± 0.04	0.462	Average fixation duration (s)	0.28 ± 0.14	0.23 ± 0.14	1.000

	Original version				Modified version		
	Document 1 (*n* = 3)	Document 2 (*n* = 3)			Document 1 (*n* = 3)	Document 2 (*n* = 3)	
	Mean ± SD	Mean ± SD	P-Value		Mean ± SD	Mean ± SD	P-Value
Getting stuck	2.00 ± 1.73	2.00 ± 1.00	0.817	Getting stuck	1.33 ± 0.58	0.33 ± 0.58	0.099
Rereading/reading twice	5.67 ± 2.08	3.00 ± 2.00	0.184	Rereading/reading twice	5.00 ± 2.65	2.00 ± 1.73	0.268
Reading one character at a time/inserting pauses	7.33 ± 6.51	4.33 ± 2.52	0.658	Reading one character at a time/inserting pauses	5.67 ± 2.08	2.67 ± 0.58	0.046*
Skipping over	4.67 ± 2.08	1.67 ± 0.58	0.046*	Skipping over	1.67 ± 1.15	1.67 ± 0.58	0.814
Reading in interrogative form	3.33 ± 3.06	2.67 ± 1.53	0.658	Reading in interrogative form	0.33 ± 0.58	0.67 ± 1.15	0.796
Misreading	13.67 ± 3.51	2.00 ± 2.00	0.050*	Misreading	3.33 ± 1.15	2.33 ± 1.53	0.346

Mann–Whitney U test was used

*Statistically significant at *p* < 0.05

Fixation count and regression are reported as frequencies. Reading time and average fixation duration are measured in seconds

Table 3 shows comparisons between participants who read aloud and those who read silently. The left column presents results for the original text, and the right column for the modified version. No significant differences were observed between the two groups. However, participants who read silently demonstrated lower average values for reading time, fixation count, and regression compared with those who read aloud.

**Table 3:** Comparison between participants who chose to read aloud and those who chose to read silently

	Original version				Modified version		
	Reading aloud (*n* = 6)	Reading silently (*n* = 3)			Reading aloud (*n* = 6)	Reading silently (*n* = 3)	
	Mean ± SD	Mean ± SD	*P*-Value		Mean ± SD	Mean ± SD	*P*-Value
Reading time (s)	287.70 ± 159.38	185.07 ± 24.13	0.606	Reading time (s)	193.30 ± 96.02	139.57 ± 96.92	0.606
Fixation count	574.50 ± 493.11	414.67 ± 46.52	0.606	Fixation count	440.33 ± 450.15	310.00 ± 272.46	0.606
Regression	182.50 ± 156.06	129.00 ± 27.62	0.796	Regression	132.33 ± 139.57	107.67 ± 73.91	1.000
Average fixation duration (s)	0.23 ± 0.13	0.32 ± 0.10	0.197	Average fixation duration (s)	0.25 ± 0.16	0.29 ± 0.10	0.439

Mann–Whitney U test was used

*Statistically significant at *p* < 0.05

Fixation count and regression are reported as frequencies. Reading time and average fixation duration are measured in seconds

Table 4 presents the comparison between the original and modified versions based on eye movement analysis during reading. For all participants (Table 4, left), “reading time” was significantly shorter for the modified version than for the original (*p* = 0.015). Participants were then separated into those who read aloud and those who read silently, and analyses were conducted within each group. For participants who read aloud (Table 4, center), the modified version produced significant reductions in “reading time” (*p* = 0.028), “fixation count” (*p* = 0.046), and number of “regression” (*p* = 0.046) compared with the original. For participants who read silently (Table 4, right), no significant differences were observed; however, the modified version showed lower average values for reading time, total fixations, and regression compared with the original. (Insert Table 4)

**Table 4:** Comparison between the original and modified versions

	All (*n* = 9)			Reading aloud (*n* = 6)			Reading silently (*n* = 3)		
	Original	Modified		Original	Modified		Original	Modified	
	Mean ± SD	Mean ± SD	*P*-Value	Mean ± SD	Mean ± SD	*P*-Value	Mean ± SD	Mean ± SD	*P*-Value
Reading time (s)	253.49 ± 136.59	175.39 ± 93.98	0.015*	287.70 ± 159.38	193.30 ± 96.02	0.028*	185.07 ± 24.13	139.57 ± 96.92	0.285
Fixation count	521.22 ± 398.62	396.89 ± 386.59	0.066	574.50 ± 493.11	440.33 ± 450.15	0.046*	414.67 ± 46.52	310.00 ± 272.46	0.285
Regression	164.67 ± 127.00	124.11 ± 117.01	0.086	182.50 ± 156.06	132.33 ± 139.57	0.046*	129.00 ± 27.62	107.67 ± 73.91	0.593
Average fixation duration (s)	0.26 ± 0.13	0.26 ± 0.14	0.110	0.23 ± 0.13	0.25 ± 0.16	0.345	0.32 ± 0.10	0.29 ± 0.10	0.109

Wilcoxon signed-rank test was used

* Statistically significant at *p* < 0.05

Fixation count and regression are reported as frequencies. Reading time and average fixation duration are measured in seconds

Table 5 shows the comparison of the original and modified versions based on the analysis of participants’ reading aloud characteristics. The modified version resulted in a significant reduction in “reading in interrogative form” (*p* = 0.041) relative to the original. Although the other measures did not differ significantly, the modified version consistently produced lower average values than the original.

**Table 5:** Comparison between the original and modified versions among participants who read aloud

	Reading aloud only (*n* = 6)		
	Original	Modified	
	Mean ± SD	Mean ± SD	*P*-Value
Getting stuck	2.00 ± 1.26	0.83 ± 0.75	0.058
Rereading/reading twice	4.33 ± 2.34	3.50 ± 2.59	0.128
Reading one character at a time/inserting pauses	5.83 ± 4.71	4.17 ± 2.14	0.293
Skipping over	3.17 ± 2.14	1.67 ± 0.82	0.108
Reading in interrogative form	3.00 ± 2.19	0.50 ± 0.84	0.041*
Misreading	7.83 ± 6.88	2.83 ± 1.33	0.115

Wilcoxon signed-rank test was used

*Statistically significant at *p* < 0.05

All metrics are expressed as frequency counts

### Qualitative analysis of participants’ reading behavior

The results of the qualitative analysis of participants’ reading are shown in Table 6 for the original version and Table 7 for the modified version. Major categories are indicated 【 】, categories by < >, and details in *italics*.

**Table 6:** Content observed when participants read the documents: Original version

Major categories	Categories	Doc
Behaviors before reading starts	Adjusts paper position for easier reading	◇◆
	Touching the eye tracker	◇◆
Variations in reading methods and styles	Chooses reading method (reads aloud or silently)	◇
	Does not hold the paper while reading	◆
	Checks the back and entire document again	◇◆
	While reading silently, says “yes” aloud in response to each sentence or context	◆
	Reads aloud only certain parts (e.g., bullet point numbers)	◆
	Change the reading of numbers (e.g., calendar years, monetary amounts)	◆
Desired reading support	Check the task (reading) with the caregiver	◇◆
	Checks with the caregiver regarding reading and where to start reading	◇◆
Respond to caregiver’s reading support	Points to the reading position when caregiver asks	◆
	Begins reading after understanding caregiver’s instructions	◆
	Repeats readings taught by caregiver	◇
Self-describe about difficulties of reading	States uncertainty about how or where to start reading	◇
	Tells caregiver, “I cannot read it.”	◆
Reading strategies	Adjusts oral reading speed (e.g., slows down, pauses)	◇◆
	Points to text to maintain reading position	◇◆
Fixation eye movements	Exhibits long-distance eye movement at line breaks	◇
Adaptability in reading	Searches for and returns to the reading position after losing it	◇◆
	Continues reading despite errors	◇◆
	Rereads words or sentences correctly after comprehension	◇◆
	Reads smoothly (e.g., across line breaks, kanji, technical terms, parentheses)	◇◆
	Discusses document content	◇◆
	Tells caregiver that reading is complete	◇◆
Errors in reading aloud	Misreads (e.g., kanji, technical terms, line breaks)	◇◆
	Word skipping	◇◆
	Loses reading position	◇
	Attempts multiple reading strategies but still misreads (e.g., pauses, questioning tone, rereads)	◇◆
	Stops reading when unable to proceed	◇
	Shows inconsistent oral reading of the same word	◆
Difficulty utilizing reading support	Responds identically to caregiver’s support each time	◆
	Unable to understand caregiver’s reading support	◇

Doc: Indicates the document in which each category was observed

◇◆ Common to Documents 1 and 2,

◇ Only Document 1

◆ Only Document 2

**Table 7:** Content observed when participants read the documents: Modified version

Major categories	Categories	Doc
Behaviors before reading starts	Adjusts paper position for easier reading	◇◆
	Touching the eye tracker	◇◆
Variations in reading methods and styles	Chooses reading method (reads aloud or silently)	◇
	Does not hold the paper while reading	◆
	Checks the back and entire document again	◇◆
	Nodding at each sentence	◆
	Change the reading of numbers (e.g. western calendar years and monetary amounts)	◆
Desired reading support	Check the task (reading) with the caregiver	◇◆
	Checks with the caregiver regarding reading and where to start reading	◇◆
Respond to caregiver’s reading support	Begins reading after understanding caregiver’s instructions and explanations	◇◆
Self-describe about difficulties of reading	States uncertainty about how or where to start reading	◇
	Tells caregiver, “I can read it, but I cannot remember it.”	◆
	Tells caregivers “I cannot read it.”	◇
Reading strategies	Adjusts oral reading speed (e.g., slows down, pauses)	◇◆
	Points to text to maintain reading position	◇◆
Fixation eye movements	Exhibits long-distance eye movement at line breaks	◇
Adaptability in reading	Discusses document content	◇
	Continues reading despite errors	◇
	Reads smoothly (e.g., across line breaks, kanji, technical terms, parentheses)	◇◆
	Rereads words or sentences correctly after comprehension	◇
	Searches for and returns to the reading position after losing it	◇
	Tells caregiver that reading is complete	◇◆
	Reads smoothly in the modified version, even in parts that were difficult to read in the original version	◆
Errors in reading aloud	Loses reading position	◇
	Word skipping	◇◆
	Misreads (e.g., kanji, technical terms, line breaks)	◇◆
	Tries rereading, but misreads	◆
	Shows inconsistent oral reading of the same word	◆

Doc: Indicates the document in which each category was observed

◇◆ Common to Documents 1 and 2,

◇ Only Document 1

◆ Only Document 2

The original version yielded 10 major categories, whereas the modified version produced nine. Both versions included the following:【Behaviors before reading starts】, 【Variations in reading methods and styles】, 【Desired (Requiring) reading support】, 【Response to caregiver’s reading support】, 【Self-description of reading difficulties】, 【Reading strategies (skills)】, 【Fixation eye movements】, 【Adaptability in reading】, and 【Errors in reading aloud】. 【Difficulty in utilizing reading support】 appeared only seen in the original version. The details are shown below.

*The caregiver instructs the participant*,* who cannot read the kanji in the technical terms*,* that “you can skip over them”. However*,* the participant does not skip them and instead asks the caregiver*,* “How do you read this?” The caregiver provides the correct reading*,* but the participant does not understand it. (omission)… (Mild dementia*,* female)*.

This major category was not found in the modified version results, and no major categories appeared exclusively in the modified version. The following sections present the categories derived from the qualitative analysis of participants’ document reading. These categories include content observed only in either the original or modified version of the analysis. These are limited to those common to Documents 1 and 2. Categories found only in the original text analysis included < Searches for and returns to the reading position after losing it>, < Continues reading despite errors>, < Reread words and sentences correctly after comprehension>, < Discusses document content> in 【Adaptability in reading】. In addition, < Attempt various reading strategies but misread (e.g., pause, read in a questioning tone, reread, etc.) > in 【Errors in reading aloud】. The details are shown below.

*He struggles with line breaks and katakana but continues reading without pausing.* *(Mild dementia, male)*

*She reads place names in the document as “place, ..” and looks at the caregiver, asking, “We went to, right?”* *(Mild dementia, female)*

In contrast, no categories were found exclusively in the modified version. 

## Discussion

We first analyzed eye movements of individuals with dementia while reading documents to identify factors that influenced or did not influence differences related to document type (Documents 1 and 2), reading method (aloud or silent), and revision status (original or revised). Based on these findings, we explored strategies to improve document readability. Subsequently, we examined factors contributing to differences between the original and revised versions in a qualitative analysis of reading comprehension, with the aim of identifying approaches to support document reading.

### Differences based on document type (comparison of documents 1 and 2)

Analysis of eye movements during document reading showed that Document 2 had fewer instances of “reading time,” “fixation count,” “regression,” and “average fixation duration” than Document 1. Previous studies indicate a relationship between saccade amplitude and fixation time: as saccade amplitude increases, the proportion of saccades decreases, whereas fixation time increases [31]. Furthermore, as text difficulty increases, fixation time lengthens, accompanied by increases in forward and regression saccades [13]. Taken together, these findings suggest that documents with shorter “time spent reading” (the sum of total fixation time and saccade time), fewer saccades, and longer fixation times are easier to read. Accordingly, Document 2 appeared less difficult to read than Document 1 for older adults with dementia. Document 2 also had a lower readability score and fewer characters than Document 1. The findings of this study suggest that reducing document difficulty and word count can lessen the time required for word and sentence processing, decrease cognitive load, and mitigate attention deficits. These results indicate that such modifications may be effective strategies for enhancing document readability. Simplifying content and shortening character count appear to lower processing demands and reduce the influence of attention deficits, thereby improving ease of reading. The number of saccades can be determined as “total number of fixations – 1.” In this study, however, saccades were not calculated; instead, the total number of fixations was recorded. Additionally, the number of “readbacks,” which constitute a portion of saccades, was counted.

On the other hand, the average fixation duration also decreased. During fixation, new information is incorporated into the processing system. Word characteristics affect fixation time: longer words extend fixation, whereas higher-frequency words shorten it [32]. Japanese writing combines kanji (ideographic characters) with two types of kana (syllabic characters). Kanji are fixated on more often than hiragana, katakana, or punctuation marks [33]. These findings suggest that short, frequent words reduce the difficulty of lexical processing. Accordingly, we predicted that average fixation duration would be shorter in Document 2 than in Document 1, and shorter in the revised version than in the original. However, no significant difference was observed. This suggests that fixation duration may be influenced not only by word length, frequency, and document difficulty, but also by reading style (aloud or silently) and document content. Skipping or refixating words complicates measurement of word processing time, and using average fixation duration is considered inappropriate because it underestimates total eye time on words [6]. In this study, average fixation duration was calculated without distinguishing between initial fixation and rereading. Therefore, it cannot serve as a reliable indicator of word processing time, and further verification is needed.

### Differences depending on how you read (comparison of reading aloud and silent reading)

We compared eye movement measurements during reading aloud and silent reading. Although no significant differences were found between the two modes in either the original or revised text, participants who read aloud spent more time reading, made more fixations, and showed more regressions than those who read silently. Previous studies of Japanese reading in adults without dementia have reported inconsistent findings when comparing aloud and silent reading. This study’s results suggest that focusing on accurate oral reading may have increased fixations and regressions to ensure precise recognition of each character. The distinction between reading aloud and silent reading will be discussed further in the next section.

### Whether or not the document has been revised (comparison of the original and revised versions)

Finally, we compared eye movement measurements according to whether the document had been revised. Among participants who read aloud, reading time, fixation count, and regressions were significantly lower in the revised text than in the original. In addition, the frequency of “reading in interrogative form” significantly decreased. One characteristic of reading aloud is that the constraints associated with speech are believed to enhance concentration and reduce the effects of attention deficits [34]. Reading aloud also increases reading time and reduces the extraction of peripheral visual information [35]. These findings suggest that participants may have improved uncertain reading by directing greater attention to the text to ensure accurate oral reading. The short-term memory enhancement effect of reading aloud, combined with the restriction of eye movements through coordination of voice and eye movement, may have heightened attention to the words and prevented loss of meaning or position within the text. As a result, significant decreases were observed in reading time, fixation count, regressions, and interrogative reading. In this way, effective use of reading aloud appears to improve eye movements during document reading. Among participants who read silently, the revised text showed decreases in reading time, fixation count, and regressions compared with the original, but these differences were not significant. This reduction suggests that revision improved readability, but because the benefits of oral reading were absent, no significant changes were detected.

In this study, the percentage of kanji was 39.7% in the original text of Document 1 and 38.8% in Document 2, compared with 27.2% and 30.4% in the revised versions, respectively. The revised version of Document 1 had the lowest proportion of kanji usage. Reports indicate that saccade distance in mixed kanji–kana text is approximately 3.2 characters [36]. Although simple calculations are not possible because some words contain multiple kanji characters, words beginning with kanji tend to draw visual attention. Therefore, placing kanji every three to four characters in a sentence is likely to produce optimal saccades. In the revised version of Document 1, the relatively low kanji proportion may have hindered the formation of appropriate saccades, potentially impeding improvements in eye movements such as reduced fixation count and regression. The percentage of kanji cannot be generalized because it does not reflect character readability and frequency of use; however, attention to the percentage of kanji characters is advisable to ensure appropriate saccades.

Based on these findings, document revision—such as adjusting difficulty, character count, number of lines, number of kanji characters, font size, and character and line spacing—combined with reading aloud without time constraints, may enhance document readability for older adults with dementia.

### Reading approaches and assistance from others

When comparing the major categories of the original and revised texts based on categories extracted from reading behavior, the major category 【Difficulty in utilizing reading support】 appeared only in the original text. To examine the factors contributing to this difficulty, it is first necessary to define “support.” This study referred to King’s A Theory of Goal Attainment, a well-known nursing theory [37]. According to this theory, nursing is “a process of human interaction between nurses and clients, in which each person perceives the other and the situation in which they are placed, sets goals through communication, explores means, and agrees on means for achieving goals.” In the context of reading documents, interaction occurs not only between nurses and patients with dementia, but also among care staff and family members. Therefore, the term “nursing” in this theory was replaced with “care and support.” Based on this interpretation, support can be defined as an interactive process between the supporter and the recipient aimed at achieving shared goals. Communication is understood as the activity or process of expressing thoughts and feelings or providing information to others [38]. Because this study focuses on document comprehension, verbal communication was emphasized. Common symptoms of language and communication disorders in dementia include word-finding difficulty, use of indirect expressions, impaired auditory comprehension, apraxia of speech, grammatical and writing disorders, loss of meaning and coherence in conversation, and difficulty adhering to social communication norms [2]. Age-related declines in speech perception have also been reported. A study on speech recognition under speech-on-speech masking conditions showed that older adults’ performance significantly decreased in the presence of masking [39]. These findings suggest that older adults may struggle to distinguish target speech in environments with multiple voices. Drawing on prior research, the 【difficulty in utilizing reading support】 observed in the original text may be associated with reduced concentration, short-term memory impairment, limited auditory comprehension, and difficulty hearing speech associated with background noise or competing voices. The original text contained technical terms and infrequently used kanji characters, suggesting that the written and spoken information provided by support staff were not effectively integrated.

Next, we considered ways to support individuals with dementia in reading documents. The broad categories 【Respond to caregiver’s reading support】 and 【Adaptability in reading】 appeared in the original and revised versions. In 【Respond to caregiver’s reading support】, specific observations included < Begins reading after understanding caregiver’s instructions and explanations > and < Repeats readings taught by caregiver>. These findings suggest that individualized pointing and verbal communication effectively promote reading behavior when appropriately tailored to each person. This is consistent with communication support strategies for patients with dementia reported in previous studies [40], such as using meaningful gestures and pointing, structurally simple sentences, and familiar words.

Furthermore, we examined how documents related to older adults with dementia were read, based on categories common across all texts. In 【Behaviors before reading starts】, < Touching the eye tracker > suggested that unfamiliar situations, such as wearing an eye tracker, may have influenced attention and concentration. In 【Desired reading support】, one participant asked the staff whether they should begin reading, as in < Check the task (reading) with the caregiver>. When handing out the paper, staff instructed participants to “read it”; however, some may not have responded because of short-term memory impairment, difficulty understanding verbal instructions, or anxiety in an unfamiliar environment. These observations indicate that, when older adults with dementia read documents, it is essential to create an environment that supports concentration and to provide assistance not only with reading but also with initiating the reading process. In 【Reading strategies】, common techniques such as pointing to the text while reading and adjusting reading speed were observed. Because no control group was established, further research is required to determine whether these behaviors are characteristic of older adults with dementia. In 【Adaptability in reading】, individual differences were noted depending on the document. Some participants < Continues reading despite errors>, whereas others stopped at unreadable sections and struggled to continue. These differences likely reflect variations in reading experience and the degree of attention deficit disorder among participants. Additionally, the participant who < Talk about the contents of the document > appeared to show interest in the material, possibly influenced by individual differences in interest and engagement.

Based on these findings, it is suggested that when supporting older adults with dementia in reading documents, it is important to assess dementia severity, background, reading ability, and the extent of communication difficulties. Individualized support should involve adjusting reading materials, creating an environment conducive to concentration, and carefully planning the timing and content of verbal cues.

### Limitations

This study has several limitations. First, the level of understanding of the document content was not confirmed; therefore, the relationship between changes in eye movements—resulting from differences between the original and revised texts, or between reading aloud and silent reading—and document comprehension could not be examined. Furthermore, we were unable to provide conclusive evidence that participants who chose silent reading were actively engaging in comprehension. Second, although improvements in eye movements were observed in the revised document, the inclusion of multiple revisions made it impossible to identify which changes were most effective. In particular, since this study focuses on Japanese, the complexity of the language makes it difficult to specify concrete improvement measures in these results, which remains a topic for future consideration. Third, the small sample size and absence of a control group limit the generalizability of the findings. Additionally, differences in the male-to-female ratio across documents may have influenced the results. Consequently, it cannot be concluded that the observed outcomes represent the general characteristics of older adults with dementia. Despite these limitations, this study remains valuable in clarifying changes in eye movements and reading progress among older adults with dementia. Future research should assess comprehension levels, examine the specific impact of each document revision, and explore support strategies that incorporate nonverbal communication.

## Conclusions

Eye movements were measured using the original and revised versions of documents for older adults with dementia. The revised documents produced significant changes in eye movements, suggesting that the revisions had a positive effect. This improvement was more evident among participants who chose to read aloud. Effective support for document comprehension in older adults with dementia requires not only document revision but also environmental adjustments and individualized assistance aligned with each person’s communication abilities.

## Supplementary Information

Supplementary Material 1.

## Data Availability

All data generated or analyzed during this study are included in this published article.

## References

[1] World Health Organization (WHO). Dementia. https://www.who.int/news-room/fact-sheets/detail/dementia. Accessed Sep 2025.

[2] Krein L, Jeon YH, Amberber AM. Development of a new tool for the early identification of communication-support needs in people living with dementia: an Australian face-validation study. Health Soc Care Community. 2020;28(2):544–54. 10.1111/hsc.12887. 31670440 10.1111/hsc.12887

[3] Murphy J, Oliver T. The use of Talking Mats to support people with dementia and their carers to make decisions together. Health Soc Care Community. 2013;21(2):171–80. 10.1111/hsc.12005. 23095078 10.1111/hsc.12005

[4] Nakamura K, Meguro K, Yamazaki H, Ishizaki J, Saito H, Saito N, et al. Kanji-predominant alexia in advanced Alzheimer’s disease. Acta Neurol Scand. 1998;97(4):237–43. 10.1111/j.1600-0404.1998.tb00644.x. 9576638 10.1111/j.1600-0404.1998.tb00644.x

[5] Humphreys GW, Bruce V. Visual cognition: computational, experimental, and neuropsychological perspectives. 2021. 10.4324/9781315785141.

[6] Starr MS, Rayner K. Eye movements during reading: some current controversies. Trends Cogn Sci. 2001;5(4):156–63. 10.1016/s1364-6613(00)01619-3. 11287269 10.1016/s1364-6613(00)01619-3

[7] Rayner K. Eye movements and attention in reading, scene perception, and visual search. Q J Exp Psychol (Hove). 2009;62(8):1457–506. 10.1080/17470210902816461. 19449261 10.1080/17470210902816461

[8] Rayner K. Eye movements in reading and information processing: 20 years of research. Psychol Bull. 1998;124(3):372–422. 10.1037/0033-2909.124.3.372. 9849112 10.1037/0033-2909.124.3.372

[9] Hannonen S, Andberg S, Kärkkäinen V, Rusanen M, Lehtola JM, Saari T, Korhonen V, Hokkanen L, Hallikainen M, Hänninen T, Leinonen V, Kaarniranta K, Bednarik R, Koivisto AM. Shortening of saccades as a possible easy-to-use biomarker to detect risk of Alzheimer’s disease. J Alzheimers Dis. 2022;88(2):609–18. 10.3233/JAD-215551. 35662117 10.3233/JAD-215551 PMC9398059

[10] Mosimann UP, Felblinger J, Ballinari P, Hess CW, Müri RM. Visual exploration behaviour during clock reading in Alzheimer’s disease. Brain. 2004;127(2):431–8. 10.1093/brain/awh051. 14691059 10.1093/brain/awh051

[11] Pelak VS. Ocular motility of aging and dementia. Curr Neurol Neurosci Rep. 2010;10(6):440–7. 10.1007/s11910-010-0137-z. 20697981 10.1007/s11910-010-0137-z

[12] Fernández G, Mandolesi P, Rotstein NP, Colombo O, Agamennoni O, Politi LE. Eye movement alterations during reading in patients with early Alzheimer disease. Invest Ophthalmol Vis Sci. 2013;54(13):8345–52. 10.1167/iovs.13-12877. 24282223 10.1167/iovs.13-12877

[13] Lueck KL, Mendez MF, Perryman KM. Eye movement abnormalities during reading in patients with Alzheimer disease. Neuropsychiatry Neuropsychol Behav Neurol. 2000;13(2):77–82. 10780625

[14] Yong KXX, Rajdev K, Shakespeare TJ, Leff AP, Crutch SJ. Facilitating text reading in posterior cortical atrophy. Neurology. 2015;85(4):339–48. 10.1212/WNL.0000000000001782. 26138948 10.1212/WNL.0000000000001782 PMC4520813

[15] Rimkeit SB, Claridge G. Literary Alzheimer’s: a qualitative feasibility study of dementia-friendly book groups. figshare. Dataset. 2018. 10.6084/m9.figshare.5715052.v2

[16] Nagarajan K, Ravi K, Periakaruppan SP, Satgunam P. When noise becomes signal: A study of blink rate using an eye tracker. Ocul Surf. 2024;34:516–20. 10.1016/j.jtos.2024.11.002. 39491645 10.1016/j.jtos.2024.11.002

[17] Tobii Pro. Tobii Pro Glasses 2 user manual v2.0.2. p41. https://go.tobii.com/Glasses2UM. Accessed Sept 2025.

[18] Legge GE, Pelli DG, Rubin GS, Schleske MM. Psychophysics of reading—I. Normal vision. Vision Res. 1985;25(2):239–52. 10.1016/0042-6989(85)90117-8. 4013091 10.1016/0042-6989(85)90117-8

[19] Akutsu H, Legge GE, Ross JA, Schuebel KJ. Psychophysics of reading—X. Effects of age-related changes in vision. J Gerontol. 1991;46(6):P325–31. 10.1093/geronj/46.6.p325. 1940088 10.1093/geronj/46.6.p325

[20] Brennan A, Worrall L, McKenna K. The relationship between specific features of aphasia-friendly written material and comprehension of written material for people with aphasia: an exploratory study. Aphasiology. 2005;19(8):693–711.

[21] Worrall L, Rose T, Howe T, Brennan A, Egan J, Oxenham D, McKenna K. Access to written information for people with aphasia. Aphasiology. 2005;19(10–11):923–9. 10.1080/02687030544000137.

[22] National Institute of Advanced Industrial Science and Technology (AIST). Database of sensory characteristics of older persons and persons with disabilities. https://scdb.db.aist.go.jp/. Accessed Sept 2025.

[23] jReadability PORTAL. https://jreadability.net/. Accessed Sept 2025.

[24] Oakland T, Lane HB. Language, reading, and readability formulas: implications for developing and adapting tests. Int J Test. 2004;4(3):239–52. 10.1207/s15327574ijt0403_3.

[25] Dale E, Chall JS. The concept of readability. Elem Engl. 1949;26(1):19–26.

[26] Slattery TJ, Rayner K. The influence of text legibility on eye movements during reading. Appl Cogn Psychol. 2010;24(8):1129–48.

[27] Osaka N. Reading: information processing in the brain and mind. Tokyo: Asakura Shoten; 1998.

[28] Braun V, Clarke V. Using thematic analysis in psychology. Qual Res Psychol. 2006;3(2):77–101. 10.1191/1478088706qp063oa.

[29] Sambai A, Coltheart M, Uno A. The effect of the position of atypical character-to-sound correspondences on reading Kanji words aloud: evidence for a sublexical serially operating Kanji reading process. Psychon Bull Rev. 2018;25(2):498–513. 10.3758/s13423-018-1434-9. 29404800 10.3758/s13423-018-1434-9

[30] Leong CK, Tamaoka K. Use of phonological information in processing Kanji and katakana by skilled and less skilled Japanese readers. Read Writ. 1995;7(4):377–93.

[31] Zihl J. Eye movement patterns in hemianopic dyslexia. Brain. 1995;118(4):891–912. 10.1093/brain/118.4.891. 7655887 10.1093/brain/118.4.891

[32] Fernández G, Castro LR, Schumacher M, Agamennoni OE. Diagnosis of mild Alzheimer disease through the analysis of eye movements during reading. J Integr Neurosci. 2015;14(1):121–33. 10.1142/S0219635215500090. 25728469 10.1142/S0219635215500090

[33] Kajii N, Nazir TA, Osaka N. Eye movement control in reading unspaced text: the case of the Japanese script. Vision Res. 2001;41(19):2503–10. 10.1016/s0042-6989(01)00132-8. 11483180 10.1016/s0042-6989(01)00132-8

[34] Miller SD, Smith DEP. Differences in literal and inferential comprehension after reading orally and silently. J Educ Psychol. 1985;77(3):341–8. 10.1037/0022-0663.77.3.341.

[35] Adedeji VI, Vasilev MR, Kirkby JA, Slattery TJ. Return-sweep saccades in oral reading. Psychol Res. 2022;86(6):1804–15. 10.1007/s00426-021-01610-6. 34694488 10.1007/s00426-021-01610-6 PMC9363329

[36] Sainio M, Hyönä J, Bingushi K, Bertram R. The role of interword spacing in reading japanese: an eye movement study. Vision Res. 2007;47(20):2575–84. 10.1016/j.visres.2007.05.017. 17697693 10.1016/j.visres.2007.05.017

[37] King IM. A theory for nursing: systems, concepts, process. New York: J Wiley; 1981.

[38] Oxford Advanced Learner’s Dictionary. com · mu · ni · ca · tion (n.). JapanKnowledge. https://japanknowledge.com/lib/display/?lid=4008000695600 Accessed Sept 2025.

[39] Helfer KS, Freyman RL. Aging and speech-on-speech masking. Ear Hear. 2008;29(1):87–98. 10.1097/AUD.0b013e31815d638b. 18091104 10.1097/AUD.0b013e31815d638b PMC2987598

[40] May AA, Dada S, Murray J. Identifying components of a person-centered augmentative and alternative communication intervention for people with dementia: opinions of an international expert panel. Am J Speech Lang Pathol. 2024;33(4):2067–82. 10.1044/2024_AJSLP-23-00317. 38901000 10.1044/2024_AJSLP-23-00317

