# Supplementary material for: Eye movement and reading behavior in older adults with dementia
Source: BMC Geriatr. 2026 Jan 29;26:206. doi: 10.1186/s12877-026-07030-8 (PMC12896137; doi:10.1186/s12877-026-07030-8)
Supplement: Supplementary file 1 — Supplementary Material 1. [file 12877_2026_7030_MOESM1_ESM.docx]

Interview Questions

After measuring the first eye movement, an interview was conducted using the following questions.

- When do you typically read documents?
- When reading documents, which parts do you find difficult to read?
- How difficult is it for you to read documents?
- How do you cope with these difficulties?

After the second measurement of eye movements, an interview was conducted using the following questions.

- Has the readability changed in the modified version compared to the original version?
- (If there were changes) How did it change compared to the original version?
- Did you find any particular parts of the modified version especially easy to read?
- Did you find any particular parts of the modified version difficult to read?
